# A novel thermochromic myocardial phantom for radiofrequency ablation and cryoablation

**DOI:** 10.1038/s41598-025-18732-1

**Published:** 2025-10-07

**Authors:** Carlo Saija, Sukruth Pradeep Kundur, Lisa Leung, Sachin Sabu, Marco Pinto, Mark Herridge, Adharvan Gabbeta, Rashi Chavan, Nadia M Chowdhury, Gregory Gibson, Calum Byrne, Antonia Pontiki, Richard James Housden, Pierre Berthet-Rayne, Jonathan M. Behar, Kawal Rhode

**Affiliations:** 1 School of Biomedical Engineering and Imaging Sciences, King’s College London, London, UK; 2 Caranx Medical, Nice, France; 3 St. George’s Hospital, London, UK; 4 St Thomas Hospital, London, UK; 5 Biosense Webster, Irvine, USA; 6 3IA Côte d’Azur, Université Côte D’Azur, Nice, France

**Keywords:** Cardiology, Engineering, Materials science, Physics

## Abstract

In the growing field of electrophysiology, cardiac ablation either via heating or freezing target tissues using catheters, induces localised scarring to block or destroy defective electrical pathways in the heart. To test equipment and ablation settings, studies have resorted to ex-vivo and in-vivo models including pigs, dogs, and chickens. The use of animal tissue presents ethical and logistical complications and introduces variability between samples and between studies. In a scientific community faced with progressively more stringent ethics and regulations on animal testing, a more practical and ethical alternative should be established. To meet this need, multiple studies have proposed tissue-mimicking materials. However, either toxicity or poorly matched physical properties, prevented these materials from reaching widespread application. Furthermore, no material has yet been identified to test both cryoablation and radiofrequency ablation. Here, we present a novel thermochromic alginate hydrogel material that can simulate ablation lesions for both radiofrequency ablation and cryoablation. This material could find direct applications in electrophysiology, but adapted mixtures could also be used to recreate other tissues for different simulations.

## Introduction

Cardiac ablation (CA) has become a well-established procedure to treat cardiac arrhythmias, which affect a growing number of patients worldwide^1,2^. Radiofrequency cardiac ablation (RCA) uses high temperatures (50–60 °C)^3,4^ delivered by a catheter to induce localised scarring in the arrhythmogenic regions of the heart. Radiofrequency ablation finds applications across a plethora of medical procedures, e.g. the treatment of liver tumours^5^ and gastroesophageal reflux disease^6^. An alternative to RCA often utilised in pulmonary vein isolation is cryoablation. Low temperature between − 30 °C and − 80 °C is applied to freeze cells and create areas of controlled scar^7–9^.

As a minimally invasive procedure yielding effective results in tackling the growing demand for arrythmia treatment, there is great potential in the development of CA technology, and subsequently, a significant need for standardised testing and training. Currently, the majority of clinical studies^4,10–12^ tested RCA using ex-vivo tissue models, mainly myocardial and skeletal muscle acquired from swine, dogs, and occasionally chickens. Due to the nature of freezing tissue, ex-vivo muscle does not visibly respond to cryoablation. To analyse the effects of this procedure, in-vivo tests are performed on dogs^8,9,13^ and the histological effects are analysed post-mortem, days to months after. As well as ethical, financial, and practical difficulties presented by in-vivo and ex-vivo testing, the non-homogeneity of tissue samples poses difficulties in testing^14,15^. In CA, the locus of tissue acquisition (e.g. ventricular wall, cardiac septum, skeletal muscle of the thigh), and the testing apparatus in which the samples are placed, can affect lesion shape and size. Post-operative lesion visualisation, in addition to the plasticity and structural uniformity of the tissue, pose limitations to reliable data acquisition. As a result, numerous studies propose myocardium-mimicking materials for ablation testing.

For the myocardium-mimicking material to be suitable, it must display realistic thermal conductivity, realistic electrical resistivity, a realistic mechanical response to the catheter, and must show visibly measurable change post-ablation. Albumin^16^ and thermochromic inks^17,18^ have been introduced in conductive polyacrylamide gel to create a conductive medium that could be ablated and would permanently change colour in the areas that reached RCA temperatures (≥ 50 °C). This was applied to RCA as well as liver ablation to assess the ablation lesion dimensions. Reversible thermochromic liquid crystals found similar applications in polyacrylamide^19,20^, allowing the users to simulate radiofrequency ablation and produce full thermal maps. Despite its repeated appearance in literature, safety concerns regarding the preparation of polyacrylamide complicate the production of all its tissue-mimicking derivates^17^. The neurotoxicity of the acrylamide monomer^21^ and its correlation with cancer mean that its preparation and handling must be carried out in a controlled environment with appropriate precautions taken. S. Wang et al.^4^ proposed electrically conductive 3D-printed cardiac models coated in 60 °C irreversible thermochromic pigment to display ablation lesions. The models raised minimal safety concerns and could be 3D-printed to simulate the shape of the heart, but RCA resulted in superficial lesions on the paint coat. Other alternatives using agar^22^, gelatine^23^, carrageenan^24^, and photopolymerised hydrogel^25^ have been proposed, but often came at the compromise of physical properties when compared to polyacrylamide. Despite the numerous materials proposed to simulate RCA, no similar research could be identified in the field of cryoablation.

Calcium alginate, a seaweed-derived hydrogel, has been cited as an ideal tissue-mimicking material due to its tough cross-linked structure and high water-content^26–28^. Fast preparation and minimal safety concerns^29,30^ make moulding calcium alginate an effective and practical material in this context. It presents neither the complications associated with animal tissue, nor the complicated process and safety hazards encountered when making polyacrylamide gel. Calcium alginate commonly finds applications in dentistry as a moulding material and in the food industry^31^ as a vegan ingredient, further emphasising its ethical-compliance and safety. Unlike agar, carrageen, or gelatine, moulding alginate does not melt under 100 °C; it can resist higher water temperatures without deterioration and is structurally unaffected by freezing. Similarly to polyacrylamide, the material on its own is not sensitive to radiofrequency or freezing, so ablation-sensitive pigments must be introduced into the mixture to visualise ablation lesions for analysis.

In this investigation, conductive alginate hydrogel was prepared with 3 non-hazardous pigments designed to change colour distinctly at the temperatures reached in RCA (50–60 °C) and cryoablation (− 40– − 80 °C). The resulting non-hazardous tissue-mimicking hydrogel formulations realistically replicate all the previously mentioned physical properties with < 15 min preparation time. This gel is the first practical, ethical, and safe alternative to in-vivo and ex-vivo models to simulate both RCA and cryoablation.

## Results

### Alginate formulation

**Fig. 1:**
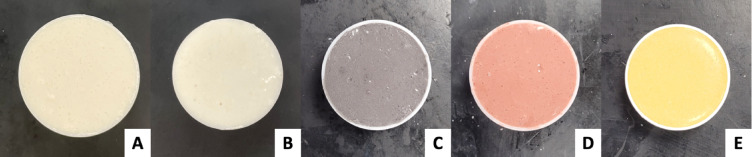
(**A**) C25 alginate mixture. (**B**) C30 Lure. (**C**) SFXC alginate mixture. (**D**) NNC alginate mixture. (**E**) Kromagen alginate mixture.

Figure 1 shows the appearance different alginate mixtures: the control mixtures have a similar consistent white colour, while the coloured mixtures acquired the grey, red and yellow tones form their pigments. Preparation time was within 15 min for each mixture after having all components measured. All alginate batches cost less than £3.00 to make. A batch of either control mixture cost £0.37, whereas a batch of SFXC, NNC, and Kromagen cost £2.09, £2.15 and £2.09 respectively.

### Electrical resistivity

Control mixtures C25 and C30 displayed electrical resistivities of respectively 12.1 ± 0.4 Ωm and 10.0 ± 0.4 Ωm. The SFXC, NNC, and Kromagen mixtures each displayed resistivities of 7.4 ± 0.6 Ωm, 7.1 ± 0.2 Ωm, and 7.0 ± 0.5 Ωm, respectively. Three-day-old ex-vivo porcine myocardium displayed an electrical resistivity of 7.6 ± 0.6 Ωm in C. Saija et al.^32^. All the coloured alginates were less resistive than the respective control mixtures. No statistically significant difference was detected at the 5% level between SFXC and heart (*p* = 0.5131), NNC and heart (*p* = 0.0520), or Kromagen and heart (*p* = 0.0548).

### Hardness

The C25 and C30 mixtures respectively showed 23 ± 3 and 23 ± 2 OO Shore hardness. The SFXC mixture displayed 27 ± 2 OO Shore hardness. It was significantly harder than the C30 mixture (*p* = 0.0014) and significantly harder than the literature-based reference (*p* < 0.0001). The NNC mixture displayed 21 ± 2 OO Shore hardness. It was significantly softer than the C30 mixture (*p* = 0.0391) and showed no statistically significant difference against the literature-based reference (*p* = 0.4004). The Kromagen mixture displayed 19 ± 2 OO Shore hardness. It was significantly softer than the C25 mixture (*p* = 0.0391) and showed no statistically significant difference against the literature-based reference (*p* = 0. 4936).

### Thermal conductivity

The average TC of SFXC alginate was 0.67 ± 0.02 W/m K, which showed no significant difference with the literature overall average TC (*p* = 0.1840). The average TC of NNC alginate was 0.62 ± 0.02 W/m K, which showed no significant difference with the TC of ventricular wall measured at 1.5 W (*p* = 0.8686). The average TC of Kromagen alginate was 0.58 ± 0.05 W/m K, which showed no significant difference with the TC of ventricular wall measured at 0.5 W (*p* = 0.9439).

**Fig. 2:**
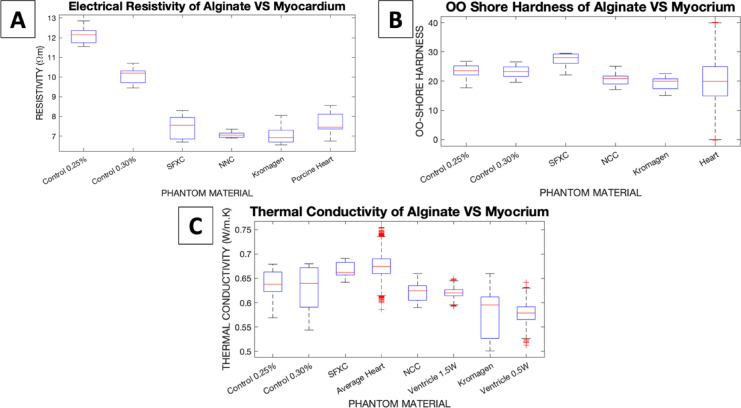
Physical properties of alginate mixtures compared to literature-derived references. (**A**) Electrical resistivity of alginate compared to ex-vivo porcine myocardium in the same experimental set-up^32^. (**B**) OO Shore hardness of alginate compared to myocardium as reported in (**A**). Tejo-Otero et al.^38^. (**C**) Thermal conductivity of alginate compared to ex-vivo porcine myocardium thermal conductivity values derived from D. Končan et al.^15^. Red crosses show outliers.

Electrical conductivity, Shore hardness, and thermal conductivity results are show graphically in Fig. 2.

## Radiofrequency ablation

**Fig. 3:**
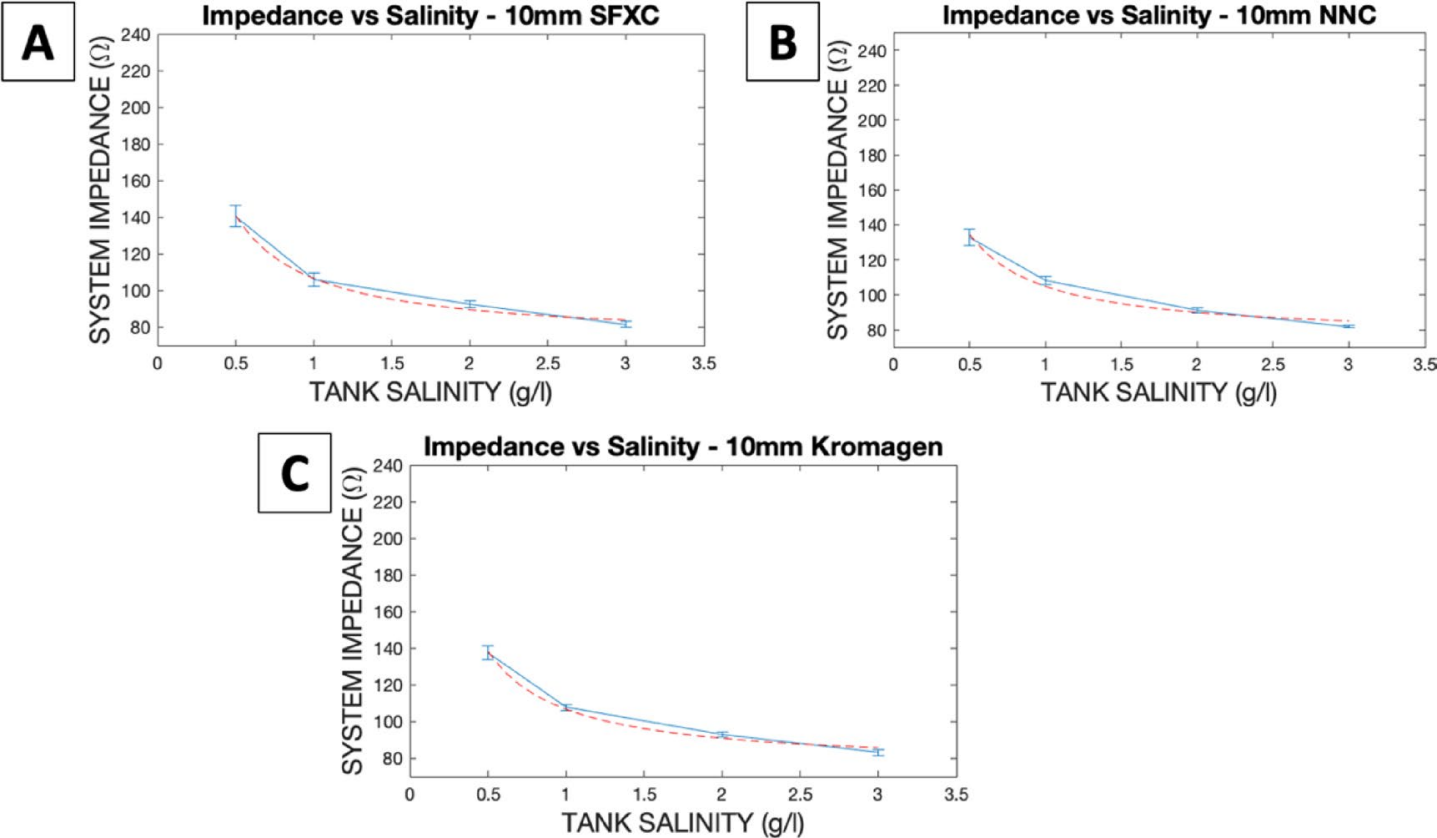
System impedance measured on 10 mm SFXC (**A**), NNC (**B**), and Kromagen (**C**) alginate slices in different saline concentrations in the 55 × 38 × 27 cm tank. The dotted red line represents Eq. (1) plotted as the line of best fit to represent the relationship between Impedance and Tank Salinity.

**Fig. 4:**
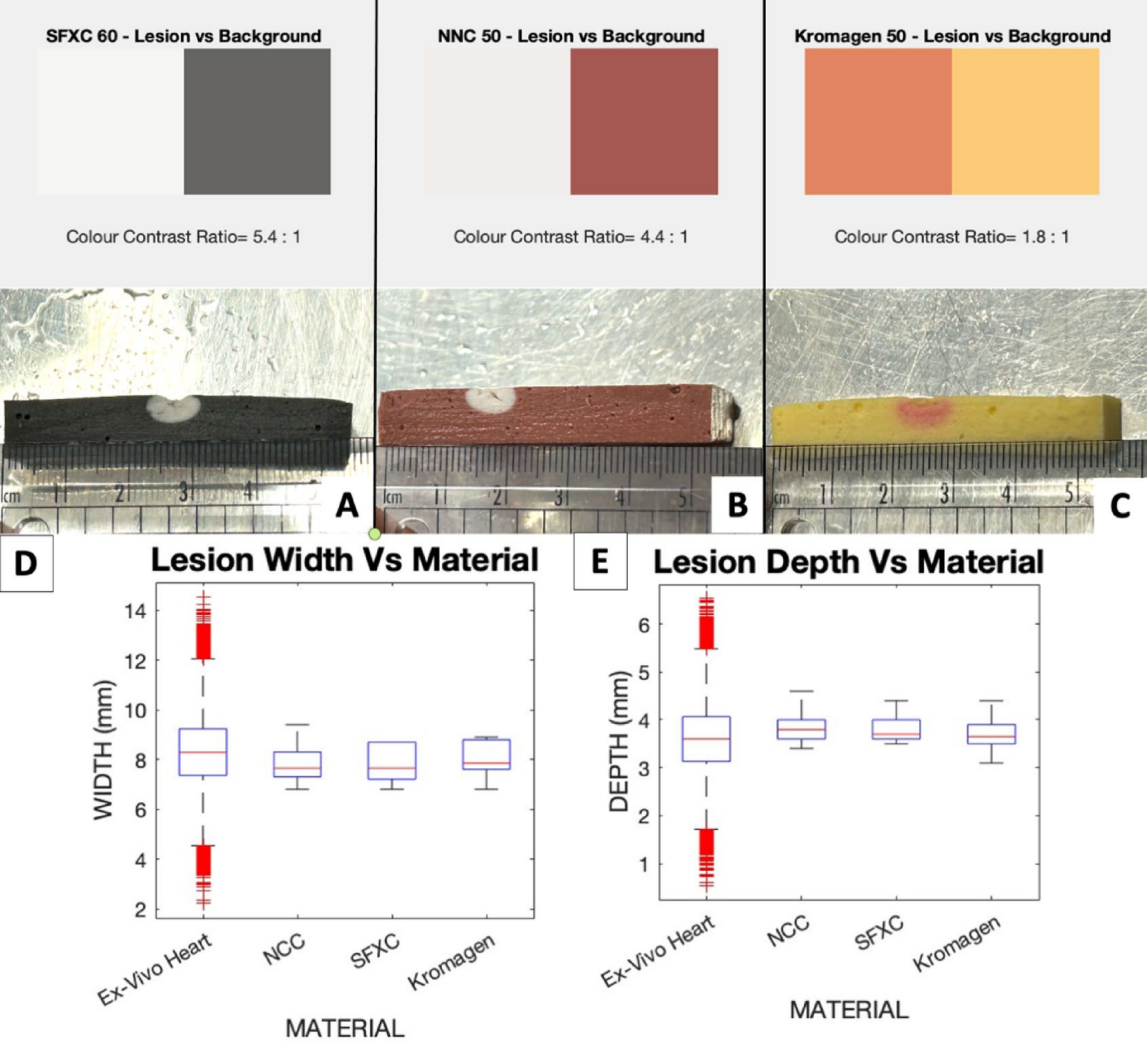
(**A**,** B**,** C**) RCA lesions shape and colour contrast for Kromagen (**A**) SFXC (**B**), NNC (**C**). (**D**) Radiofrequency ablation lesion width in coloured alginate mixtures compared to ex-vivo porcine myocardium from Barkagan et al.^11^. (**E**) Radiofrequency ablation lesion depth in coloured alginate mixtures compared to ex-vivo porcine myocardium from Barkagan et al.^11^.

System impedance for all three coloured mixtures (shown in Fig. 3) followed the expected inverse relationships with tank salinity and there was no difference in the trend followed by the three alginate mixtures. SFXC ablation lesions measured 3.8 ± 0.3 mm in depth and 7.8 ± 0.8 mm in width. SFXC lesions had no statistically significant difference in depth (*p* = 0.0565) and width (*p* = 0.0765) compared to literature values. RGB values for the lesion on SFXC alginate was (245, 246, 243), whilst background was (102, 101, 99). Colour contrast for the lesions against the background was 5.4:1. NNC ablation lesions measured 3.8 ± + 0.4 mm in depth and 7.9 ± 0.9 mm in width. NNC lesions had no statistically significant difference in depth (*p* = 0.0693) and width (*p* = 0.1929) compared to literature values. RGB values for the lesion on NNC alginate was (240, 237, 235), whilst background was (155, 92, 85). Contrast for the lesions against the background was 4.4:1. Kromagen ablation lesions measured 3.7 ± 0.4 mm in depth and 8.0 ± 0.7 mm in width. Kromagen lesions had no statistically significant difference in depth (*p* = 0.4422) and width (*p* = 0.1942) compared to literature values. RGB values for the lesion on Kromagen alginate was (214, 137, 104), whilst background was (244, 205, 131). Colour contrast for the lesions against the background was 4.4:1. All lesions had lower variance in the lesion width and lesion depth than the literature values. These results are shown graphically in Fig. 4.

### Cryoablation

**Fig. 5:**
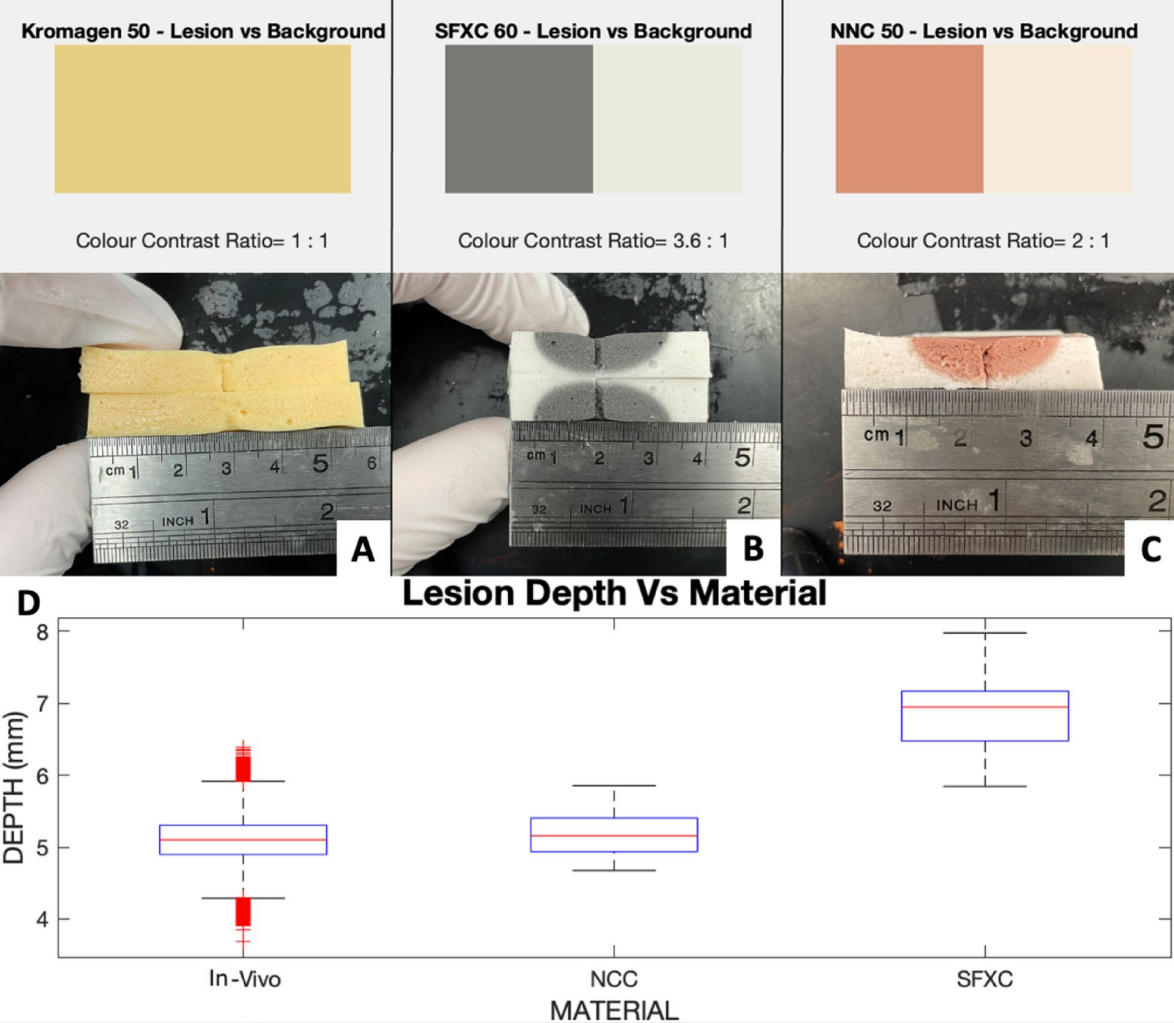
(**A**,** B**,** C**) Cryoablation lesions shape and colour contrast for Kromagen (**A**) SFXC (**B**), NNC (**C**). (**D**) Cryoablation lesion depth for coloured alginates (marked as NNC and SFXC) after cryoablation compared to in-vivo dogs from Bessière et al.^8^.

Only SFXC and NNC responded to cryoablation. After preparation, Kromagen progressively reverted back from magenta to yellow over one hour. Kromagen did not retain a permanent colour change after cryoablation. SFXC cryoablation lesions measured 6.9 ± 0.6 mm in depth. There was a statistically significant difference between SFXC lesion depth and literature values (*p* < 0.0001 in an unpaired two-tailed t-test). RGB values for the lesion on SFXC alginate was (121, 122, 113), whilst background was (234, 235, 221). Contrast for the lesions against the background was 3.6:1. NNC cryoablation lesions measured 5.2 ± 0.3 mm in depth. There was no statistically significant difference between NNC lesion depth and literature values (*p* = 0.2458 in an unpaired two-tailed t-test). RGB values for the lesion on NNC alginate was (246, 235, 219), whilst background was (208, 248, 121). Contrast for the lesions against the background was 2:1. In all cryoablations, temperature remained between − 40 °C and − 80 °C, as expected^8^. These results are shown in Fig. 5.

## Discussion

The method used to create the alginate mixtures was easy, fast, and replicable. It involved the use of non-hazardous materials, and all samples were ready within 15 min of having all the components measured. All materials costs were less than £3.00 per batch, which is comparable to the cost of ex-vivo animal tissue.

The value of resistivity measured in ex-vivo porcine myocardium for this study (7.6 ± 0.6 Ωm) is coherent with the in-vivo electrical resistivity of ventricular myocardium (7.04 ± 2.11 Ωm) reported in literature^37^, validating the method adopted in this study. All the coloured alginate samples had no statistically significant difference to porcine myocardium reported in C. Saija et al.^32^. All pigments were conductive since the addition of these significantly decreased the resistivity of the alginate in all cases. The most electrically conductive pigment was Kromagen 50, which reduced the resistivity of C25 from 12.0 ± 0.4 Ωm to 7.0 ± 0.5 Ωm. The samples were only directly compared to ventricular myocardium from C. Saija et al.^32^. To provide a broader comparison, similar readings could have been recorded for different tissues in the heart, such as pericardial fat or connective tissue; however, for the purpose of this study, the electrical resistivities were accurate enough to simulate heart tissue.

NNC and Kromagen alginates had realistic Shore hardness on the OO scale and showed no significant difference against the literature-derived hardness of ex-vivo myocardium, whereas SFXC alginate was harder and showed significant difference against the reference. This may stem from SFXC being the only dry pigment used in this study, whilst NNC and Kromagen were pigment slurries. Lower water content makes alginate harder, and water absorption from the pigment may have occurred, ultimately making the sample harder. Future investigations should evaluate if the change in Shore hardness from OO 20 ± 7.5 to 27 ± 2 can be detected by operators at different levels of experience, and if it significantly effects the force response on the catheter during navigation.

SFXC (0.67 ± 0.02 W/m K) had the highest TC and showed no statistically significant difference with the overall average TC from ex-vivo myocardium from literature (0.675 ± 0.02 W/m K). Kromagen (0.58 ± 0.05 W/m K) showed no statistically significant difference with ex-vivo ventricular myocardium measured at 0.5 W (0.58 ± 0.02 W/m K). Whereas NNC (0.62 ± 0.02 W/m K) showed no statistically significant difference with ex-vivo ventricular myocardium measured at 1.5 W (0.62 ± 0.01 W/m K). Because all results matched literature values of TC from ex-vivo porcine myocardium, all mixtures had appropriately realistic TC for the purpose of a simulation. The method utilised in this test was accurate, however, the standard deviation of TC from the literature values was lower. Future studies should aim to investigate the thermal permittivity as well as the heat capacity of these hydrogels to further simulate heat dissipation post-ablation, insulating the apparatus to improve precision.

In the case of radiofrequency ablation, the method directly replicated the one implemented in Barkagan et al.^11^, using the same CARTO 3 (Biosense Webster, Irvine, USA) set up and settings. All coloured mixtures showed no statistically significant difference when compared to the lesion dimensions achieved in Barkagan et al.^11^ at on ex-vivo myocardium, thus all mixtures could be used to effectively simulate RCA. The pigmented mixtures produced greater precision with comparable means to the literature, emphasising the improvement in results replicability that can be achieved from this hydrogel. Kromagen and NNC are more sensitive, changing colour at 50 °C, while SFXC changes colour at 60 °C but has higher TC. Consequently, no significant difference in lesion dimensions was identified amongst the three mixtures. SFXC and NNC had the best colour contrasts at 5.4:1 and 4.4:1, making the boundary of the lesions well defined and easy to notice. Kromagen, on the other hand, is a pigment which gradually changes colour and had a colour contrast of 1.8:1, ultimately making the ablation lesion less defined than in SFXC and NNC. This behaviour could be subject of future studies to assess temperature gradients created during RCA. Unexpectedly, Kromagen alginate lesions faded in colour over time, posing a potential limitation to the widespread use of this pigment. Further research would uncover more of the colour behaviour of Kromagen and its potential application in RCA. Though this investigation analysed up to a single pigment per mixture, future developments may combine multiple thermochromic agents of different sensitivities to produce colourful thermal maps indicating the various temperatures reached within the alginate matrix.

In the case of cryoablation, our experiment simulated the methods performed on dogs in Bessière et al.^8^ using the Medtronic Artic Front Advance (Medtronic CryoCath LP, Montreal, Canada) and CryoCath console (Medtronic CryoCath LP, Montreal, Canada) using the same console the same ablation settings. The NNC mixture resulted in ablation lesions comparable to the literature. SFXC responded to freezing, however, the lesions were too large. This discrepancy may be attributed to the difference in TC as well as the pigment’s sensitivity to freezing. Kromagen did not generate any lesion after freezing and did not maintain colour after heating, making it unusable for this simulation. The colour contrast between lesion and background was lower after cryoablation than after RCA, indicating the pigments may be more sensitive to heat. Due to the shape of the catheter, the only measure that could be compared was depth. Future investigations should aim to replicate this test using a range of different catheters to compare to literature while also moulding the material in the shape of atria to test for lesion-transmutability and oesophageal injury. Though only incident in 1% of patients, atrio-oesophageal fistula formation ensuing from CA results in an 80% mortality rate^33^. This material may therefore find extensive applications in modern research in this field^34,35^, allowing operators to study tissue damage and temperature transfer between different layers of tissue.

All hydrogels achieved high-fidelity to ex-vivo myocardium and produced realistic results under RCA, but only NNC performed realistically during cryoablation. Assessing the performance across all tests, the NNC mixture obtained the best results overall, having found no statistically significant difference against ex-vivo myocardium on any of the physical tests, while displaying realistic lesions dimensions. Despite its unique colour changing properties, Kromagen had a low colour contrast and underperformed during cryoablation, ultimately making it not a good option. Its properties could be further investigated to produce data on heat propagation in the gel. The SFXC mixture obtained the best electrical properties, thermal properties, and colour contrast, however its hardness was significantly higher than myocardium and it was outperformed by NNC in cryoablation. Ultimately, SFXC performed better than Kromagen but not NNC. In future investigations, users may employ 3D-printing or moulds to shape the alginate mixtures into heart phantoms which could also be perfused with water pumps to emulate blood flow and create a highly-realistic simulation environment without using animal tissue.

Though in-vivo tests still remain the most realistic test conditions, the NNC thermochromic hydrogel hereby formulated arguably outperformed ex-vivo porcine myocardium, as it could markedly show the ablation lesion for both cryoablation and RF ablation. This gel is the first synthetic model that allows operators to compare multiple ablation modalities (such as RCA or cryoablation), new ablation equipment (such as catheters), and ablation techniques (such as single shot ablation or point-by-point) without immediately resorting to live animal studies.

To conclude, the study assessed the performance of three tissue-mimicking hydrogels for cardiac ablation simulation. The results indicate that the NNC alginate mixture can reliably replicate the thermal, electrical, and mechanical properties of ex-vivo porcine myocardium in order to be used to simulate response to ablation. Unlike previously attempted formulations in the literature, this material is non-hazardous, inexpensive, and ethically compliant, as well as homogeneous and replicable, making it an easy, quick, and ethical tissue-mimicking hydrogel for both RCA and cryoablation tests.

## Methods

### Alginate formulation

White calcium alginate moulding powder (Pebeo, Marseille, France) was utilised as the gelling agent. Saline was prepared at 0.3% and 0.25% mass concentrations using deionised water and NaCl. Kromagen50 (SpotSee, Dallas, Texas), SFXC 60 °C Irreversible Thermochromic Pigment (Good Life Innovations Ltd., Seaford, United Kingdom) and NNC TM-SL W50-0 Brown (New Prismatic Enterprise Co., Taipei, Taiwan) were identified as the three thermochromic pigments for these experiments as they retain their new colour after changing. Two control mixtures and three coloured mixtures were prepared following the ratios shown in Table 1. Liquids and powders were mixed and measured separately before being combined with a hand blender for 30 s. The blender was then opened, and the sides were scraped. The mixture was blended for 10 more seconds and poured in moulds to set for 10 min.

**Table 1:** Formulations for the mixtures of alginate analysed.

	Mixture	Saline mass concentration	Pigment mass (g)	Mass saline (g)	Mass alginate powder (g)
Control mixtures	Control 0.25% (C25)	0.250%	0.00	120	32.0
	Control 0.3% (C30)	0.300%	0.00	120	32.0
Coloured Mixtures	SFXC	0.300%	2.00	120	32.0
	NNC	0.300%	2.50	120	32.0
	Kromagen	0.250%	2.50	120	32.0

### Electrical resistivity

**Fig. 6:**
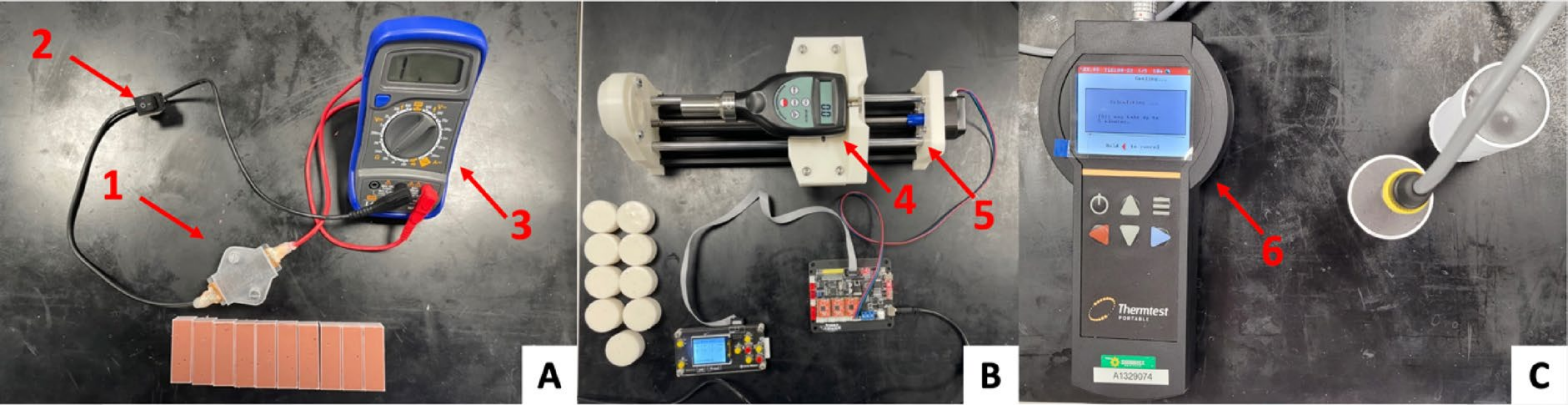
(**A**) Volume-constrained box (1) connected to a multimeter (3) using wires and a switch (2) to measure electrical resistivity. (**B**) HT-6510OO OO-shore durometer (4) mounted on the linear actuator (5). (**C**) TSL-100 thermal conductivity analyser (5) setup.

To evaluate the electrical properties of all mixtures, a volume constrained box 45 × 15 × 15 mm was 3D-printed in Anycubic Basic Clear Resin (HongKong Anycubic Technology Co., Hong Kong, China). 15 mm square copper electrodes were constructed using 0.5 mm copper sheets and placed on either side of the box. The electrodes were connected to a switch and a multimeter to measure electrical resistance. Internal resistance of the system was measured at 0.00 ± 0.00 kΩ, using the 20 kΩ range. Ten 45 × 15 × 15 mm slices of each alginate mixture were prepared and moulded to size. Each slice was placed in the box and compressed by the lid, ensuring full contact with the electrodes and consistent volume (Fig. 6A). The switch was turned on to inspect the initial resistance of the material in kΩ. Resistivity of each material was calculated by multiplying the value of resistance by the cross-sectional area of the sample, divided by its length. Electrical resistivity can otherwise be calculated as the inverse of electrical conductivity. Using the same methods and set up^32^, our previous research measured the electrical resistivity of porcine myocardium to be 7.6 ± 0.6 Ωm. In literature, D. Miklavčič et al.^36^ describes ex-vivo heart tissue to have electrical conductivity of 0.06–0.4 S/m (resistivity = 2.5–16.7 Ωm); K. Raghavan et al.^37^ reports that in-vivo myocardium has an electrical conductivity of 0.142 ± 0.043 S/m (resistivity = 7.04 ± 2.11 Ωm).

### Hardness

A HT-6510OO OO-Shore Durometer (Landtek Instruments Co., Guangzhou, China) was mounted on a remote-controlled linear actuator. On the opposite side of the actuator, a 1 mm thick cup containing a 20 × 35 mm (length x diameter) cylinder of alginate was held in place by two M4 screws, holding it centred with the durometer (Fig. 6B). The durometer was displaced by 15 mm allowing the alginate to come in to contact with the body of the durometer. 10 measurements of hardness were recorded for each mixture, using 10 separate cylinders. A. Tejo-Otero et al.^38^ reported that hydrogels of OO Shore hardness 20 ± 7.5 can be used to recreate ex-vivo cardiac muscle. Based on this, a 10,000-element vector was created following a normal distribution with mean 20 and standard deviation 7.5 using MATLAB function “normrnd” as a literature-derived reference for statistical analysis.

### Thermal conductivity

Thermal Conductivity (TC) was analysed to compare the thermal behaviour of alginate to ex-vivo myocardium, which would directly affect lesion dimensions via the propagation of heat/cold. The TC of myocardium falls between 0.58 and 0.75 W/m K^15,39^. The paper by D. Končan et al.^15^ was chosen as a reference for this investigation, as it analysed the TC of the septum and the ventricular wall, two common ablation sites. The study used varying power (0.5–1.5 W) to heat ventricular and septal ex-vivo porcine myocardium and measured their TC. The study reported ventricular TC of 0.58 ± 0.02 W/m K when heating at 0.5 W, and 0.62 ± 0.01 W/m K when heating at 1.5 W. The overall average TC of myocardium across all methods in D. Končan et al.^15^ is 0.675 ± 0.02 W/m K. Based on the means and standard deviations of these three TC values, three normally distributed arrays of 10,000 elements were generated using MATLAB function “normrnd” to provide some literature-derived references for statistical analysis.

To measure the thermal conductivity of alginate, long cylindrical 110 × 37.5 mm samples were cast inside of polylactic acid 3D-printed moulds. The TSL-100 thermal conductivity analyser (Thermtest Instruments, New Brunswick, Canada) was fully inserted into the sample and a value of thermal conductivity was measured (Fig. 6C). The test was repeated 10 times on different samples for all mixtures. Like all other measured properties, the TC values acquired during this study were compared against the reference using a 5% confidence level in a two-tailed multi-variance t-test.

### Radiofrequency ablation

**Fig. 7:**
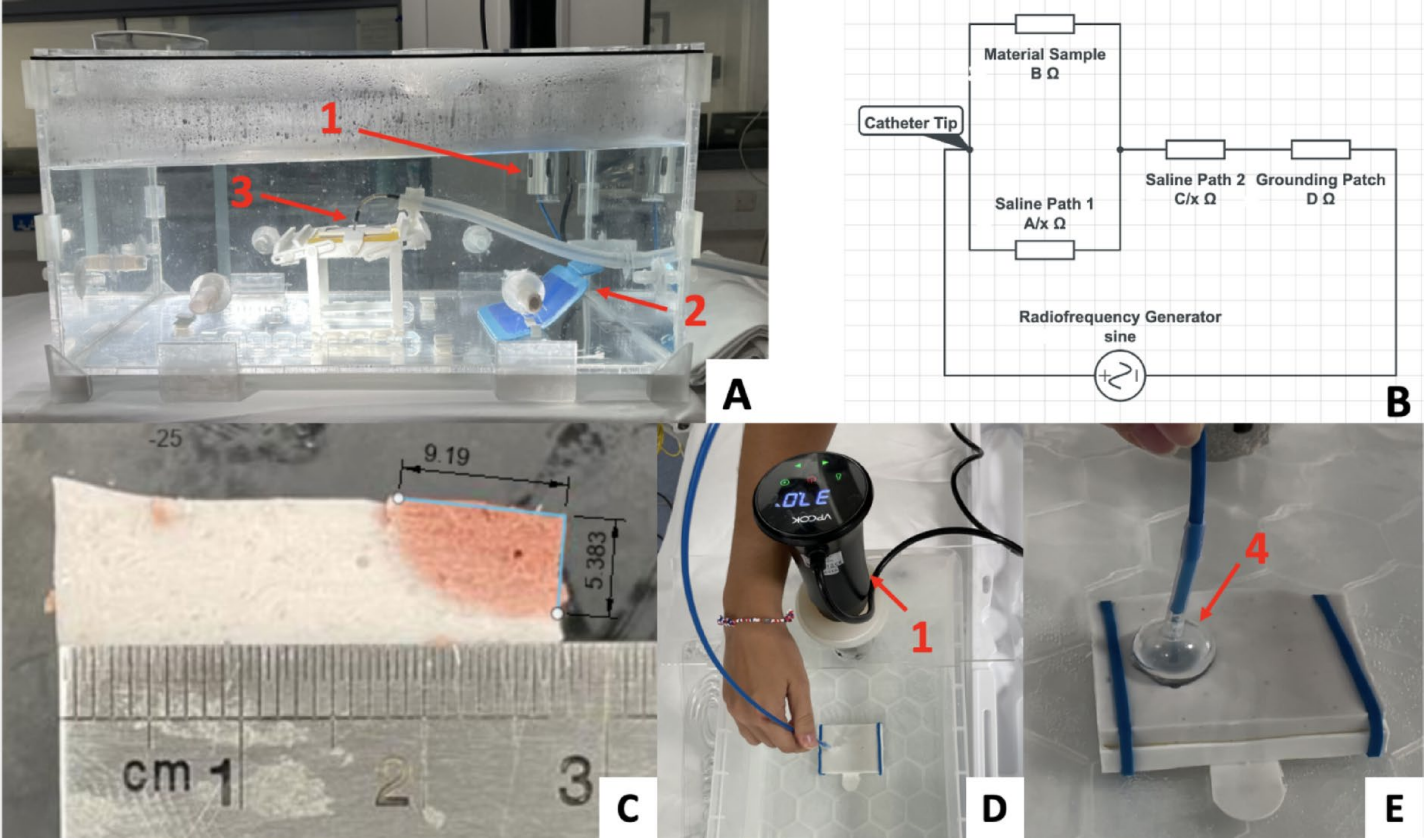
(**A**) The RCA simulator system. The thermostat system (1), Grounding patch (2), and RCA catheter (3) (**B**) Electrical path followed by the radiofrequency starting at the tip of the catheter on the left and terminating with the grounding patch on the right, derived from Barkagan et al.^11^. (**C**) Digital lesion dimensions measurements. (**D**,** E**) The cryoablation simulation system using a cryoballoon catheter (4).

To analyse the radiofrequency ablation behaviour of the hydrogel and directly compare the finding to Barkagan et al.^11^, the simulator system published in C. Saija et al.^32^ was adapted to replicate the same ablation conditions. To emulate the heart walls 75 × 55 × 10 mm slices of coloured alginate were moulded to simulate the thickness of ventricular walls^40^. The coloured alginate slices were mounted on a perforated base with a 1.5 cm sponge using a plastic frame. The base was mounted in the centre of a temperature-controlled 55 × 38 × 27 cm acrylic tank filled with saline at different concentrations. The Valleylab Return Patient electrode (Covidien, Dublin, Ireland) was placed on the bottom of the tank facing into the saline in the inferior section of the tank to simulate patch placement on the lower back of the patient (Fig. 7.A). The saline was kept at 37.0 °C and saline mass concentration was progressively increased (0.05%, 0.1%, 0.2%, 0.3%) and the baseline impedance was recorded on 5 separate locations on the slice using a Thermocool SmartTouch Unidirectional Navigation Catheter (NAV ST SF) (Biosense Webster Inc., Irvine, USA) in the CARTO VIZIGO 8.5 F (Biosense Webster Inc., Irvine, USA) steerable sheath linked to the SMARTABLATE System (Biosense Webster Inc., Irvine, USA). Based on the idealised electrical model displayed in Fig. 7B, the relationship between system impedance (R) versus tank salinity (x) can be derived as shown is Eq. 1, where A, B, C, D are constants and α = 1/A and β = 1/B.

1 $$\begin{aligned} R = & \frac{1}{{\frac{x}{A} + \frac{1}{B}}} + \frac{C}{x} + D \\ = & \frac{1}{{\alpha x + \beta }} + \frac{C}{x} + D \\ \end{aligned}$$

Barkagan et al.^11^ performed ablation lesions in a similar simulator set-up using ex-vivo porcine ventricular wall. Their low impedance (100–130Ω) tests performed lesions at 30 W for 20 s and averaged a depth of 3.6 ± 0.7 mm and a width of 8.3 ± 1.4 mm using an open-irrigated ablation catheter (Thermocool SmartTouch SF; Biosense Webster, Irvine, USA) and a SmartAblate radiofrequency generator (Stockert GmbH, Freiburg, Germany) linked to the CARTO 3 system (Biosense Webster, Irvine, USA). In order to be comparative to this published study, our tank was filled with 0.075% saline (impedance: 100–130Ω) at 37.0 °C and 10 ablation lesions were performed on 10 mm slices of the coloured alginate mixtures using the same equipment and settings. The ablation catheter was held perpendicular to the alginate sample using the steerable sheath; ablations were performed at 30 W for 20 s applying 10 g of force and irrigating with 24 °C 0.075% saline at 8 ml/min as per default. Results of lesion depth and width were measured digitally based on a ruler placed directly next to each sample as shown in Fig. 7C. Results of width and depth were compared against the findings of Barkagan et al.^11^ as previously described using MATLAB.

Finally, to evaluate the colour contrast between the lesions and the background alginate, the RGB values of the coloured mixtures were analysed based on pictures acquired under equal light conditions. The RGB values of 125 pixels were acquired from both the background and the lesions and averaged. The colour contrast was then calculated using Colour Contrast Analyser (TPGi, Clearwater, USA).

### Cryoablation

To test the cryoablation properties of the materials, the methods listed in Bessière et al.^8^ were replicated using the Medtronic Artic Front Advance (Medtronic CryoCath LP, Montreal, Canada) and CryoCath console (Medtronic CryoCath LP, Montreal, Canada) using nitrous oxide (N_2_O) refrigerant. In their experiment they cryoablated both the atrial and ventricular walls of live dogs using the same settings, resulting in mostly transmural lesions in the atrial walls (3.5 ± 0.4 mm), but not in the ventricles. More specifically, only 2.43% of left ventricular lesions were transmural due to wall thickness; the maximum lesion depth reached in this area was on average 5.1 ± 0.3 mm with exposure time 2–4 min, marking a finite limit of reachable depth. To simulate the same conditions in our experiment, two 10 mm ventricle-like alginate slices were prepared for each mixture. For colour change to occur in reverse, each mixture was prepared by adding the pigments in the saline and heating the solutions in plastic containers for 10 min at 70 °C. Each measured solution was cooled then mixed with alginate powder, as described previously, and cast into 75 × 55 × 10 mm slices. Slices were held in a 37.0 °C heated water bath; cryoablation was performed replicating the methods of Bessière et al.^8^ as closely as possible using a Medtronic Artic Front Advance (Medtronic CryoCath LP, Montreal, Canada) and the CryoCath console (Medtronic CryoCath LP, Montreal, Canada) for 3 min using nitrous oxide (N_2_O) refrigerant (Fig. 7D). As per default settings, temperatures were monitored with a sampling frequency of 10 Hz with a 1 °C accuracy maintaining temperatures within the range of − 40 °C to − 80 °C like in Bessière et al.^8^. Due to the shape of the catheter, the lesions had an annular shape (Fig. 7E). Two ablations were performed for each material. The annular ablations were sliced, and depth was digitally measured 16 times. Results of lesion dimensions were compared against the findings of Bessière et al.^8^ as previously described using MATLAB.

## Data Availability

The datasets used and/or analysed during the current study available from the corresponding author on reasonable request.
