# Correction: A novel thermochromic myocardial phantom for radiofrequency ablation and cryoablation

**DOI:** 10.1038/s41598-026-62135-9

**Published:** 2026-07-15

**Authors:** Carlo Saija, Sukruth Pradeep Kundur, Lisa Leung, Sachin Sabu, Marco Pinto, Mark Herridge, Adharvan Gabbeta, Rashi Chavan, Nadia M. Chowdhury, Gregory Gibson, Calum Byrne, Antonia Pontiki, Richard James Housden, Pierre Berthet-Rayne, Jonathan M. Behar, Kawal Rhode

**Affiliations:** 1 School of Biomedical Engineering and Imaging Sciences, King’s College London, London, UK; 2 Caranx Medical, Nice, France; 3 St. George’s Hospital, London, UK; 4 St Thomas Hospital, London, UK; 5 Biosense Webster, Irvine, USA; 6 3IA Côte d’Azur, Université Côte D’Azur, Nice, France

Correction to: *Scientific Reports* 10.1038/s41598-025-18732-1, published online 07 October 2025

The original version of this Article contained an error in the name of author Nadia M Chowdhury which was incorrectly given as Nadia Chowdry.

The original Article has been corrected.

